# Biomechanical Evaluation of the Modified Cannulated Screws Fixation of Unstable Femoral Neck Fracture with Comminuted Posteromedial Cortex

**DOI:** 10.1155/2019/2584151

**Published:** 2019-07-07

**Authors:** Jingwen Liu, Baokun Zhang, Bohao Yin, Hongchi Chen, Hui Sun, Wei Zhang

**Affiliations:** Department of Orthopedic Surgery, Shanghai Jiao Tong University Affiliated Sixth People's Hospital, Shanghai 200233, China

## Abstract

**Purpose:**

To verify the biomechanical importance with respect to the integrity of posteromedial cortex of femoral neck fracture (FNF) and demonstrate whether the modified fixation of cannulated screws (CSs) could increase the biomechanical strength.

**Methods:**

A total of 24 left artificial femurs were randomly divided into three groups. The osteotomy was made in the center of the femoral neck at a 20° angle to the shaft axial. The posteromedial cortices of femoral neck were removed in groups B and C. In group A, 8 femurs with intact posteromedial cortex were fixed with three parallel partial thread screws (PTSs), forming a standard triangle. In group B, the femurs were stabilized with the same fixation of CSs like group A. In group C, two inferior PTSs were replaced by two fully thread screws (FTSs).

**Results:**

The lower A-P and axial stiffness and load to failure along with higher axial displacement were found in group B compared with group A (p≤0.001 for all). Between groups B and C, the modified fixation of CSs increased A-P and axial stiffness and load to failure and reduced the axial displacement (p≤0.001 for all).

**Conclusions:**

We verified that the comminuted posteromedial cortex affected the biomechanical strength adversely and resulted in higher displacement. The modified fixation of CSs characterized by two inferior FTSs could improve the biomechanical performance and buttress the femoral head fragment better.

## 1. Introduction

Femoral neck fractures (FNFs) in the nonelderly population are uncommon which often result from high-energy trauma. Anatomic reduction and stable internal fixation of the femoral neck (FN) are accepted widely in an attempt to salvage the femoral head [1]. And it is the primary goal of treatment to restore the patients' normal functional mobility [2].

Multiple cannulated cancellous screws are used widely due to the advantages including less tissue invasive, less blood loss, and shorter operation time which made it a very common choice for surgeons to treat with FNF [3]. Moreover, the configuration of three parallel partially thread screws (PTSs) can be utilized to compress the fracture fragments and eliminate a potential fracture gap which can enhance fracture healing [4–7]. The use of multiple compressive screws has been advocated for Garden types 1 and 2 to promote healing [1, 8]. In the displaced FNF, however, the rate of fixation failure and femoral neck shortening increased significantly when fixed with this sliding compression device; and severe shortening had adverse effect on the functional outcome [9, 10]. Most displaced fractures (Gardens 3 and 4) are associated with posterior comminution of the femoral neck [11]. Further, Scheck early emphasized the significance of posterior comminution in femoral neck and thought it as a cause of unstable fixation [12, 13]. A literature suggested that a displaced femoral neck fracture with disrupted posterior cortex increases the risk of shortening and replacement significantly compared with the fracture with integrity of posterior cortex [14].

In view of the unstable essence of the displaced FNF with comminuted posteromedial cortex, the traditional configuration of three parallel compression screws is consistently under debate. On the premise of less invasive, we tried to introduce the fully thread screws (FTSs), which functioned as position screws, to rebuild the posteromedial buttressing effect and improve the biomechanical properties of CSs fixation.

In the present biomechanical study, we hypothesize that the improved configuration of cannulated screws could increase biomechanical strength and decrease the displacement when the posteromedial cortex was comminuted.

## 2. Materials and Methods

### 2.1. Groups of Specimens

**Figure 1:**
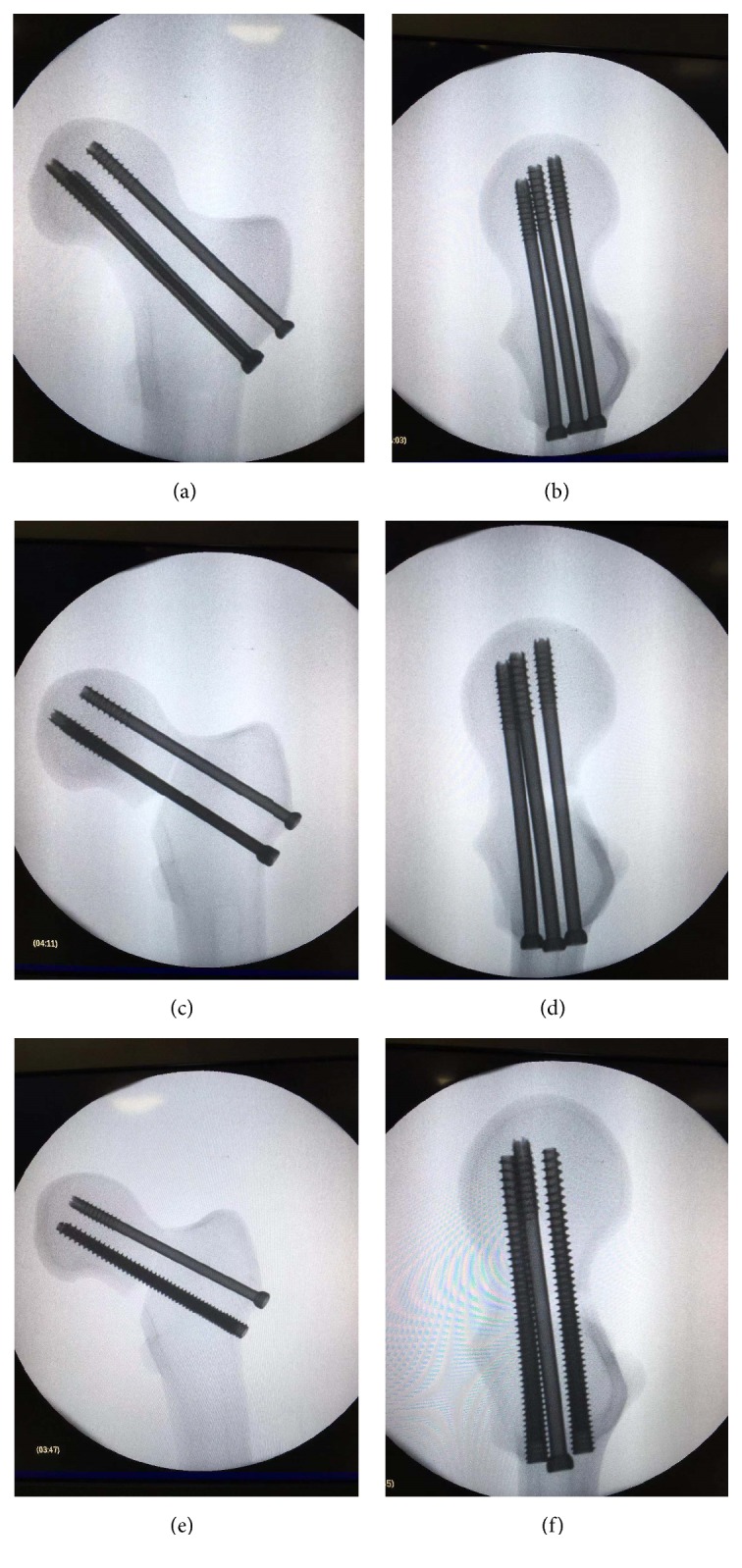
Radiographs in two planes (AP and lateral) were obtained for the three groups. (a-b) FNF with intact posteromedial cortex treated with three parallel PTSs; (c-d) FNF with comminuted posteromedial cortex treated with three parallel PTSs; (e-f) FNF with comminuted posteromedial cortex treated with one PTS and two inferior FTSs.

A total of 24 left artificial femurs (SYNBONE, Switzerland) were randomly divided into three groups (Figure 1). Group A consisted of 8 femoral neck fractures with intact posteromedial cortex fixed by three PTSs (7.3-mm cannulated screws, DePuy Synthes, Warsaw, Indiana, USA) forming standard triangle; Group B was made up of 8 specimens with comminuted posteromedial cortex treated with three parallel PTSs; Group C comprised 8 femurs with comminution of posteromedial cortex stabilized by one PTS plus two inferior parallel FTSs ( Acutrak 6/7, Acumed, Hillsboro, Oregon, USA).

### 2.2. Preparation of Specimens

**Figure 2:**
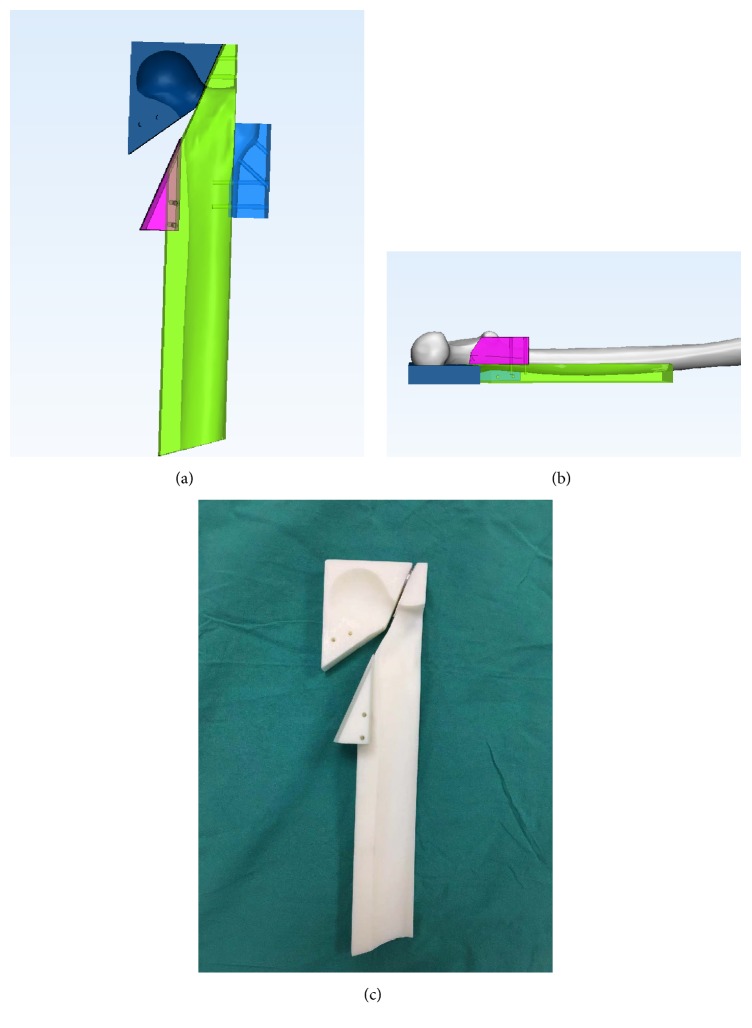
The design of mold made with 3D print technology.

To ensure consistency of all specimens, we designed a mold with 3D printing technology which mainly consisted of two modules: the main module, functioning as the pedestal and the guiding route of sawing, and the assembled module which guided the insertion of pin (Figure 2). After being placed on the main module, the femur was firstly removed from the distal third part for the better fixation. Then the osteotomy was made in the center of the neck at a 20° angle with respect to the shaft axis to simulate a Pauwels type III fracture. In groups B and C, the creation of the comminution of femoral neck posteromedial cortex was by removing two wedges: one distal wedge, cut at a 30° angle, and another posterior wedge, cut at a 15° angle, with respect to the initial osteotomy which is similar to the protocol of Windolf [15]. Subsequently, the assembled module was adhered to the main module. The first guide pin was inserted beneath the femoral neck superior cortex through the designed hole; the second was placed near anteroinferior cortex and the third one along the posteroinferior cortex; and these guide pins formed the configuration of standard triangle. The accurate placement of 3 parallel guide pins was validated through the fixed insert holes. The predrilling through guide pin was finished after removing the assembled module, and three cannulated screws were inserted in the previous order.

### 2.3. Biomechanical Testing

**Figure 3:**
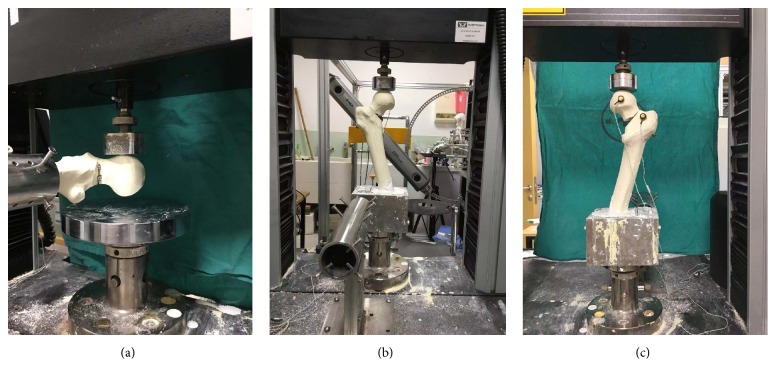
(a) The position of specimen simulating rising from the chair or climbing stairs; (b) the position of specimen simulating standing; (c) two magnets were used to measure the axial displacement.

Biomechanical testing was performed on an Instron test system (Instron, Norwood, MA, USA) which included a base, a pressure applicator, and a data analyzer. The loading protocol consisted of several parts. Firstly, to simulate the bending force when rising from the chair or climbing stairs, each specimen was stabilized in a custom-made jig distally and maintained the femoral shaft perpendicular to the load vector [16]. The specimen was preloaded to 10N at the speed of 2mm/min. Then, the load rose to 400N at the same speed and the anterior-posterior (A-P) stiffness was recorded. Secondly, the specimen was fixed in 16° adduction of the femoral shaft which was in accordance with the hip contact forces measured in vivo [17]. The distal part of femoral shaft was potted into the mental holder with dental powder. Each femur was preloaded to 10N at the above speed; next, the speed was maintained and the axial stiffness and the load to failure were calculated. Meanwhile, the length of displacement was recorded through two magnets separately adhered to the femoral head and proximal femur (Figure 3). Failure was defined as a marked decrease in the applied load value or osteotomy displacement greater than 10mm or catastrophic failure occurred.

### 2.4. Statistical Analysis

The recorded dates were analyzed with the use of SPSS software (SPSS version 20; SPSS Inc., Chicago, IL, USA). Normal distribution and homogeneity of variance were screened with Shapiro-Wilk and Levene's tests, respectively. One-way variance analysis and LSD (least-significant difference) post hoc test were performed for group comparisons. The level of significance was determined to be p<0.05.

## 3. Results

**Figure 4:**
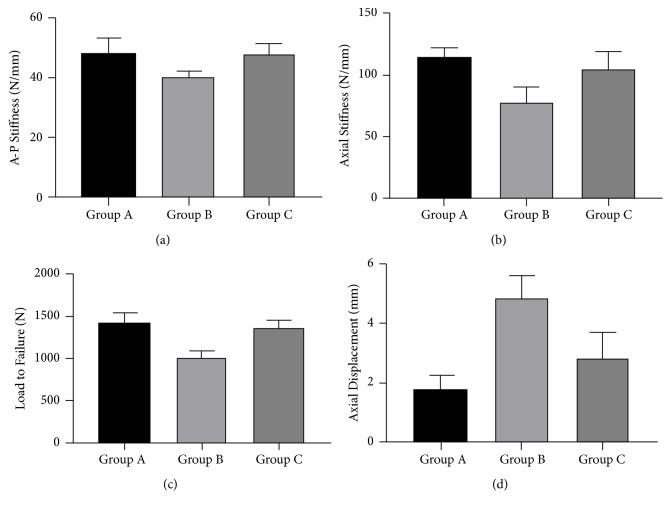
The results of the biomechanical test. (a) A-P stiffness; (b) axial stiffness; (c) load to failure; (d) axial displacement.

All results were showed as mean± standard deviation in Table 1. When the posteromedial cortex was disrupted, a significant decrease was showed in A-P and axial stiffness and load to failure, and higher axial displacement was showed (p≤0.001 for all) between groups A and B. When the two inferior PTSs were replaced by two FTSs in group C, A-P and axial stiffness and load to failure increased significantly (p≤0.001 for all). Meanwhile, axial displacement was reduced significantly. A-P and axial stiffness and load to failure showed no statistical difference comparing group A with group C; however, higher axial displacement was found in group C. The differences between two groups were entirely showed in Table 2 and Figure 4.

## 4. Discussion

In the present study, we verified that the integrity of posteromedial cortex of FN played an important role in the biomechanical fixation strength. The disruption or comminution of posteromedial cortex resulted in the significant decrease of A-P and axial stiffness, load to failure, and significantly higher axial displacement. Kauffman concluded that the femoral neck fracture with a posterior defect exhibited greater displacement in anterior loading and showed lower axial loads to failure [18]. These conclusions were comparable to ours.

The sliding compression device allowed the controlled fracture impaction across parallel placed screws to eliminate the fracture gap and increase the stability of fracture which was favorable to the fracture union [7, 19]. Nevertheless, the “controlled” impaction would be out of control and the ultimate stability was hardly achieved by sliding pressure when the posteromedial buttressing effect was lost. Some authors have regarded posteromedial femoral neck comminution as an important determinant of the fracture stability [12, 20]. Furthermore, a rate ranging from 22% to 67% in displaced fracture with the disrupted posterior cortex of FN had been reported in some literatures [12, 13, 21, 22]. Hence, we hoped to utilize the FTSs to strengthen the traditional configuration of CSs.

**Figure 5:**
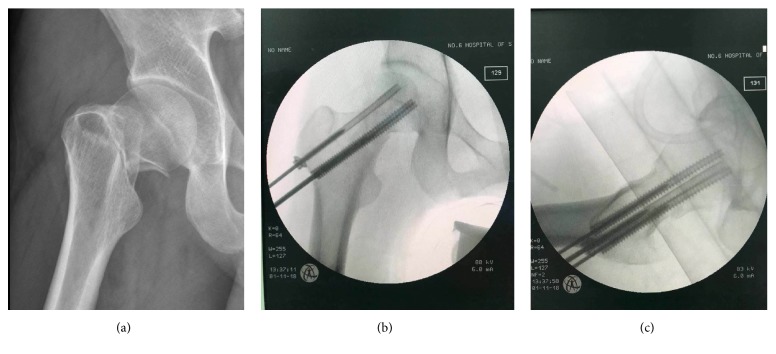
Clinical example: a 29-year-old male patient suffered a left FNF with disrupted posteromedial cortex after a motor vehicle collision. (a) The anteroposterior plain radiograph of pre-operation. (b-c) The intraoperative view after closed reduction and fixation.

In our previous study, we tried to treat the vertical femoral neck fracture with the modified configurations of CSs and the improved biomechanical and clinical outcomes verified the superior biomechanical strength to resist the shearing force and to support the femoral head fragment [23]. Hence, we hypothesized that two inferior FTSs could also be used to rebuild the lost posteromedial buttressing effect. The distal thread of FTS could grasp the lateral cortical bone as firmly as the lock-screw locked the plate. Some authors had demonstrated that the addition of a lateral locking providing angular stability of the screws may improve fracture stability and increase the resistance to shear forces in the treatment of Pauwels III fractures; the basic principle may be that they reduced the micromotion around the fracture site [24, 25]. Kuan et al. also concluded that a construct of adding a cerclage wire in combination with triangle triple cannulated screws, which greatly reduced the micromotion between the screws, can improve the biomechanical performance. The modified configuration of CSs in our study could attain the similar effect and the micromotion among the screws could be reduced due to the lock between FTSs and the lateral cortex. Schaefer et al. replaced the PTS at the 2 o'clock position with the FTS and suggested that the new construct improved the bending (A-P) stiffness and reduced the collapse of the fracture; but it was unable to increase the axial stiffness (1418±88 vs. 1479±155N/mm, p=0.3) [26]. However, a significantly higher axial stiffness in group C was showed in the present study compared with group B. The main reason could be the different methods of osteotomy. Considering the limited compression of FTSs, we firstly placed a PTS for better compression and enforced the friction between the two fracture planes; then two inferior FTSs were placed to buttress the femoral head fragment and maintain the form of proximal femur. The biomechanical improvement was showed in our new fixation of CSs for the treatment of FN with comminuted posteromedial cortex (Figure 5).

Besides the nonunion and AVN, the severe shortening of FN would be another problem facing the orthopedic surgeons. Several studies have demonstrated that the shortening of FN greater than 5mm has a negative effect on the quality of life [9, 10, 27]. In a multicentre study without the limitation of age, Zlowodzki et al. found that 30% of the fracture healed with >10mm of shortening and the rate was up to 52% in the displaced fracture treated by multiple cannulated screws [9]. Even in the young patients who mostly had a higher bone density, 21.4% of unstable FNFs experienced severe shortening (>10mm) [10]. Weil et al. had introduced the FTS to stabilize the femoral neck fracture, most of which belong to stable fracture pattern (Garden I or II) and concluded that FTSs could reduce the displacement radiographically [28]. Although the two inferior FTSs could not resist the axial displacement as firmly as the intact femoral neck cortex, the lower axial displacement was showed, compared with fixation of three PTSs.

One of the strengths in the present study is that we designed a 3D mold providing a reference system and all osteotomies and fixations were conducted under this system to minimize the operation error. Nevertheless, some limitations need to be mentioned. Firstly, the small number of samples tested would restrict the conclusion. Secondly, the use of composite bones only accounts for the young patients with good bone quality. But, for the elderly with Garden type III or IV fractures characterized by low bone mineral density, arthroplasty was preferred by most surgeons [29]. Lastly, the length of screws, which we failed to consider when purchasing, might affect the result.

## 5. Conclusions

It is not an advisable choice of traditional fixation of CSs, as three parallel PTSs, for the stabilization of the unstable FN with comminuted posteromedial cortex. The rebuilding of medial buttress with two inferior FTSs could increase the biomechanical strength and reduce the displacement. By comparison with traditional CSs fixation, three parallel PTSs, characterized by sliding compression, the modified fixation of CSs may be preferred to the treatment of unstable femoral neck fracture with comminuted posteromedial cortex.

## Figures and Tables

**Table 1:** The results of A-P and axial stiffness, load to failure, and axial displacement were showed as mean± standard deviation.

	Group A	Group B	Group C
A-P stiffness [N/mm]	48.235±5.081	40.237±2.010	48.113±3.327
Axial stiffness [N/mm]	114.463±7.112	77.239±13.231	103.813±15.342
Load to failure [N]	1422.968±110.587	1010.918±76.019	1364.580±88.389
Axial displacement [mm]	1.785±0.462	4.857±0.745	2.859±0.830

**Table 2:** The statistical difference between two groups (ANOVA with post hoc testing).

	A-P stiffness	Axial stiffness	Load to failure	Axial displacement
Group A vs B	0.000	0.000	0.000	0.000
Group A vs C	0.948	0.100	0.222	0.006
Group B vs C	0.000	0.000	0.000	0.000

## Data Availability

The data used to support the findings of this study are available from the corresponding author upon request.

## References

[1] Panteli M., Rodham P., Giannoudis P. V. (2015). Biomechanical rationale for implant choices in femoral neck fracture fixation in the non-elderly. *Injury*.

[2] Sheehan S. E., Shyu J. Y., Weaver M. J., Sodickson A. D., Khurana B. (2015). Proximal femoral fractures: what the orthopedic surgeon wants to know. *RadioGraphics*.

[3] Selvan V. T., Oakley M. J., Rangan A., Al-Lami M. K. (2004). Optimum configuration of cannulated hip screws for the fixation of intracapsular hip fractures: a biomechanical study. *Injury*.

[4] Madsen F., Linde F., Andersen E., Birke H., Hvass I., Poulsen T. D. (1987). Fixation of displaced femoral neck fractures: a comparison between sliding screw plate and four cancellous bone screws. *Acta orthopaedica Scandinavica*.

[5] Paus A., Gjengedal E., Hareide A., Jørgensen J. J. (1986). Dislocated fractures of the femoral neck treated with von Bahr screws or hip compression screw. Results of a prospective, randomized study. *Journal of the Oslo City Hospitals*.

[6] Rehnberg L., Olerud C. (1989). Fixation of femoral neck fractures comparison of the uppsala and von bahr screws. *Acta Orthopaedica Scandinavica*.

[7] Okcu G., Özkayın N., Erkan S., Koray Tosyali H., Aktuğlu K. (2015). Should full threaded compression screws be used in adult femoral neck fractures?. *Injury*.

[8] Hoskinson S., Morison Z., Shahrokhi S., Schemitsch E. H. (2015). Managing AVN following internal fixation: treatment options and clinical results. *Injury*.

[9] Zlowodzki M., Brink O., Switzer J. (2008). The effect of shortening and varus collapse of the femoral neck on function after fixation of intracapsular fracture of the hip: a multi-centre cohort study. *The Journal of Bone & Joint Surgery—British Volume*.

[10] Slobogean G. P., Stockton D. J., Zeng B., Wang D., Ma B., Pollak A. N. (2017). Femoral neck shortening in adult patients under the age of 55 years is associated with worse functional outcomes: Analysis of the prospective multi-center study of hip fracture outcomes in China (SHOC). *Injury*.

[11] Elgeidi A., El Negery A., Abdellatif M. S., El Moghazy N. (2017). Dynamic hip screw and fibular strut graft for fixation of fresh femoral neck fracture with posterior comminution. *Archives of Orthopaedic and Trauma Surgery*.

[12] Scheck M. (1980). The significance of posterior comminution in femoral neck fractures. *Clinical Orthopaedics and Related Research*.

[13] Scheck M. (1959). Intracapsular Fractures of the Femoral Neck: comminution of the posterior neck cortex as a cause of unstable fixation. *The Journal of Bone & Joint Surgery*.

[14] Huang T., Hsu W., Peng K., Lee C. (2011). Effect of integrity of the posterior cortex in displaced femoral neck fractures on outcome after surgical fixation in young adults. *Injury*.

[15] Windolf M., Braunstein V., Dutoit C., Schwieger K. (2009). Is a helical shaped implant a superior alternative to the Dynamic Hip Screw for unstable femoral neck fractures? A biomechanical investigation. *Clinical Biomechanics*.

[16] Davy D. T., Kotzar G. M., Brown R. H. (1988). Telemetric force measurements across the hip after total arthroplasty. *The Journal of Bone & Joint Surgery*.

[17] Bergmann G., Deuretzbacher G., Heller M. (2001). Hip contact forces and gait patterns from routine activities. *Journal of Biomechanics*.

[18] Kauffman J. I., Simon J. A., Kummer F. J., Pearlman C. J., Zuckerman J. D., Koval K. J. (1999). Internal fixation of femoral neck fractures with posterior comminution: a biomechanical study. *Journal of Orthopaedic Trauma*.

[19] Parker M. J., Porter K. M., Eastwood D. M., Schembi Wismayer M., Bernard A. A. (1991). Intracapsular fractures of the neck of femur. Parallel or crossed garden screws?. *The Journal of Bone & Joint Surgery (British Volume)*.

[20] Collinge C. A., Mir H., Reddix R. (2014). Fracture morphology of high shear angle 'Vertical' femoral neck fractures in young adult patients. *Journal of Orthopaedic Trauma*.

[21] Alho A., Benterud J. G., Müller C., Husby T. (1993). Prediction of fixation failure in femoral neck fractures: comminution and avascularity studied in 40 patients. *Acta orthopaedica Scandinavica*.

[22] Frangakis E. K. (1966). Intracapsular fractures of the neck of the femur. Factors influencing non-union and ischaemic necrosis.. *The Journal of Bone & Joint Surgery (British Volume)*.

[23] Zhang B., Liu J., Zhu Y., Zhang W. (2018). A new configuration of cannulated screw fixation in the treatment of vertical femoral neck fractures. *International Orthopaedics*.

[24] Basso T., Klaksvik J., Foss O. A. (2014). The effect of interlocking parallel screws in subcapital femoral-neck fracture fixation: a cadaver study. *Clinical Biomechanics*.

[25] Basso T., Klaksvik J., Foss O. A. (2014). Locking plates and their effects on healing conditions and stress distribution: a femoral neck fracture study in cadavers. *Clinical Biomechanics*.

[26] Schaefer T. K., Spross C., Stoffel K. K., Yates P. J. (2015). Biomechanical properties of a posterior fully threaded positioning screw for cannulated screw fixation of displaced neck of femur fractures. *Injury*.

[27] Stockton D. J., Lefaivre K. A., Deakin D. E. (2015). Incidence, magnitude, and predictors of shortening in young femoral neck fractures. *Journal of Orthopaedic Trauma*.

[28] Weil Y. A., Qawasmi F., Liebergall M., Mosheiff R., Khoury A. (2018). Use of fully threaded cannulated screws decreases femoral neck shortening after fixation of femoral neck fractures. *Archives of Orthopaedic and Trauma Surgery*.

[29] Bhandari M., Devereaux P. J., Tornetta P. (2005). Operative management of displaced femoral neck fractures in elderly patients. An international survey. *The Journal of Bone and Joint Surgery-American Volume*.

